# Genetic and Environmental Overlap Between Chinese and English Reading-Related Skills in Chinese Children

**DOI:** 10.1037/a0037836

**Published:** 2014-09-15

**Authors:** Simpson W. L. Wong, Bonnie Wing-Yin Chow, Connie Suk-Han Ho, Mary M. Y. Waye, Dorothy V. M. Bishop

**Affiliations:** 1 Department of Psychological Studies, The Hong Kong Institute of Education, and Department of Experimental Psychology, University of Oxford; 2 Department of Psychology, University of Oxford, and Department of Applied Social Sciences, City University of Hong Kong; 3 Department of Psychology, The University of Hong Kong; 4 School of Biomedical Sciences, The Chinese University of Hong Kong; 5 Department of Experimental Psychology, University of Oxford

**Keywords:** Chinese learners of English, second language reading acquisition, twins, behavioral genetics, cognitive–linguistic skills

## Abstract

This twin study examined the relative contributions of genes and environment on 2nd language reading acquisition of Chinese-speaking children learning English. We examined whether specific skills—visual word recognition, receptive vocabulary, phonological awareness, phonological memory, and speech discrimination—in the 1st and 2nd languages have distinct or overlapping genetic and environmental origins. A sample of 279 Chinese twin pairs with a mean age of 6 years was tested. Univariate twin analyses were used to identify sources of individual variations in reading abilities and related cognitive–linguistic skills in Chinese and English, respectively. They were used to show both similar and distinctive patterns in these skills across Chinese and English. Bivariate Cholesky decomposition analyses indicated genetic overlaps between all parallel Chinese and English variables, as well as shared environmental overlaps in receptive vocabulary and phonological awareness. The phenotypic correlations between 1st and 2nd language skills previously observed in cross-linguistic studies could be explained by the shared genetic and environmental influences found in this twin study.

Over a billion people learn English as a second language (ESL) for various purposes, making it a global phenomenon (Graddol, 2006). In addition to the variations in ESL performance across countries, substantial individual differences have been found in ESL ability within a linguistic culture.

To date, a couple of twin studies have found heritable influences on second language learning, with substantial genetic overlaps between the first and second language (Coventry et al., 2012; Dale, Harlaar, Haworth, & Plomin, 2010). These studies have examined English-speaking students learning another alphabetic language as a second language. However, the genetic overlaps between two languages with a relatively large difference in characteristics (e.g., English vs. Chinese) are yet to be documented. Also, these studies measured language ability using teachers’ ratings on students’ overall attainment. While teachers’ ratings provide useful estimates of overall language attainment, they are less suitable for assessing specific cognitive–linguistic skills such as phonological memory. Therefore, in the current study, we tested a more homogenous sample of native Chinese speakers who routinely learn English as a second language, examining the heritability of the first and second language reading skills as well as their genetic overlaps. Moreover, we employed direct measurements of reading ability as well as reading-related skills. The results have provided new insights into the nature of association between first language (L1) and second language (L2) skills.

## Phonological Skills in ESL Reading Development

Reading development in children, which involves phonological encoding by which print is mapped to sound, relies on a number of cognitive–linguistic skills. Many studies have confirmed that phonological awareness is a strong predictor of reading success (e.g., Melby-Lervåg, Lyster, & Hulme, 2012; Oakhill & Cain, 2012; Wagner, Torgesen, & Rashotte, 1994). It is further argued that accurate mental representation of speech sounds, as indicated by speech perception tasks, leads to the formation of distinct and accurate phonemic representations (Boets, Wouters, van Wieringen, De Smedt, & Ghesquiere, 2008). A series of recent studies have examined the relations among speech perception, phonological awareness, and word reading abilities, both in L1 and in L2. For example, in a study of Korean-speaking ESL children, English speech perception and phonological awareness were important contributors to early English reading abilities, independent of English oral language skills (Chiappe, Glaeser, & Ferko, 2007). As well as acquiring print-to-sound mapping, meanings of written words must be acquired. Therefore, oral vocabulary learning is a skill important to reading, as confirmed in previous studies (e.g., Bryant, MacLean, & Bradley, 1990). Moreover, vocabulary learning is supported by another important cognitive skill, namely phonological memory. Past studies have also shown a strong positive relation between phonological memory and vocabulary size (Gathercole, Hitch, Service, & Martin, 1997). Because of the strong links between reading and the aforementioned skills, our investigation focused on the genetic and environment influences on these links.

## Developmental and Cognitive Perspectives on Chinese–English Bilingual Reading Acquisition

A key question is whether proficiency in L1 helps or hinders development of L2. The two languages being examined in this study, namely Chinese and English, have enormous differences in both oral and written language systems. Broadly speaking, Chinese is logographic, while English is alphabetic. Because Chinese and English share few cognates (i.e., words that share a historical origin and have similar alphabets, spelling, and meaning across languages), Chinese ESL learners learn an independent set of English vocabulary and words in order to read English. Although there are shared consonants and vowels in the Chinese and English phonological systems, the presence of unique English phonemes usually causes perceptual difficulties in Chinese learners (e.g., A. Y. W. Chan, 2006). For example, Brown (2000) showed that Chinese learners of English were less able to discriminate /s-θ/ contrast in English (e.g., “sink” vs. “think”). A similar problem was observed in distinguishing the English front vowels /æ/and /e/ as appear in the word pair “bad–bed.” Such perceptual difficulties would hinder the formation of distinct representation of English phonemes as well as English reading. For all these reasons, one might anticipate that the better the knowledge of Chinese, the harder it might be to learn English.

However, the evidence points in the opposite direction: Research to date has shown significant positive associations between skills in the two languages. In Cheung et al.’s (2010) study, Chinese word reading, vocabulary, phonological awareness, and speech perception were significantly correlated with parallel English skills after the effects of age and nonverbal intelligence were controlled for (*r* = .26 to .58). Similar results were obtained by McBride-Chang and Ho (2005) in their study of Chinese children aged between 4 and 5. This study showed significant associations between Chinese and English skills, including word reading, phonological awareness, and verbal memory (*r* = .22 to .63). The significant relations between Chinese and English word reading were found to be stable across time (McBride-Chang et al., 2008) and were observed in Chinese children living in English-speaking countries (Bialystok, McBride-Chang, & Luk, 2005). To some extent, it could be that these correlations reflect some general task-taking ability. In general, these positive associations are important in showing that what develops is abstract knowledge, rather than just specific associations between letters or characters and sounds. Further, these findings support the idea that a portion of phonological skills is a general ability shared between the two languages, even when these languages have relatively distinct features. This fits with contemporary models of cross-linguistic transfer that maintain that transfer of skill from L1 to L2 operates at the functional level and involves a set of previously acquired meta-linguistic skills (Genesee, Geva, Dressler, & Kamil, 2006). For instance, children who have an adequate level of phonological representations and phonological processing in their L1 would develop similar proficiency in discovering the sound-to-print correspondence in another language. Similarly, as Geva (1999) has argued, if positive and significant correlations are observed between parallel meta-linguistic skills in the two languages, it is very likely that there is a common underlying cognitive factor that controls the parallel skills in two languages (see also Gholamain & Geva, 1999). This account has important implications for teaching children in two languages, because it suggests that development of literacy will not be hampered by introducing a second language: On the contrary, it is possible that learning will be facilitated by making it easier to detect abstract similarities between the languages.

## Genetic and Environmental Perspectives

The contrast between L1 and L2 is of particular interest for understanding the cognitive underpinnings of literacy development when a genetically informative twin paradigm is applied. We have noted how Chinese and English make an intriguing contrast in terms of linguistics differences. If individual differences in development depend on superficial features of the written or spoken language, we would expect very little overlap in skills in the two languages, or even some trade-offs between them. Yet evidence to date suggests the two languages support one another. An additional factor to be considered is the nature of exposure to the oral language of L1 and L2, which is the foundation for written language. Explicit training is generally thought to play little part in acquisition of the native language, and mastery of language structure proceeds on a similar trajectory without any need for formal instruction. However, English is taught to children in Hong Kong at kindergartens or schools from a very early age, and the environment for learning English is more diverse. We might therefore expect that individual differences in rate of development of L1 would reflect genetic factors, whereas for L2, environmental factors might be expected to predominate. We therefore can use the contrast between L1 and L2 to throw further light on the relative importance of genes and environment in the development of oral skills that underpin literacy.

It should be noted that the term *environment* is used in the field of behavior genetics to refer to any influence that is not genetic. Although it is possible to integrate measures of known environmental factors, such as school experiences, in a twin design, more usually, specific environmental factors are not directly assessed. Rather, the relative importance of genetic and environmental influences is estimated by comparing the degree of resemblance between monozygotic (MZ) and dizygotic (DZ) twins. Typically, twins grow up together in the same home and often attend the same school. Given the importance of environmental influences, we expect twins to resemble one another, regardless of zygosity. They differ, however, in their genetic similarity, with MZ twins being genetically identical, and DZ twins sharing, on average, 50% of their segregating genes. If MZ twins are more similar to one another than DZ twins, a genetic influence is suggested. Using this method, we can test whether there is a greater genetic influence on L1 versus L2 reading, and examine twin–cotwin similarities on a given task across languages to obtain evidence of shared genetic influence on skills in the two languages.

## The Present Study

Using a twin study design, the present study aimed to disentangle genetic and environmental influences on children’s Chinese (L1) and English (L2) skills and their associations in 279 Chinese twin pairs. There were three research questions. First, what are the relative contributions of genes and environment to individual differences in Chinese and English reading and related cognitive–linguistic skills? Univariate twin analyses were conducted to indicate the relative contributions of genetic and environmental factors to these skills. Second, are parallel Chinese and English reading skills influenced by the same set of genes and/or environments? Bivariate twin analyses were conducted to examine the overlaps between genetic/environmental factors across L1 and L2. For instance, do the genetic factors contributing to Chinese word reading overlap with those contributing to English word reading? Last, to what extent do genes and environment contribute to the Chinese–English observed phenotypic correlation? The proportions of phenotypic correlations explained by genes and environment were computed.

## Method

### Participants

The present article is a part of the Chinese Twin Study of reading development (Chow, Ho, Wong, Waye, & Bishop, 2013). The child participants were Hong Kong Chinese whose native language was Cantonese. They were recruited through schools and promotion posters. These children received compulsory education and learned to read Chinese and learned English as a second language in schools from the age of 3 years in kindergarten. All twin pairs lived together in the same household. The zygosity status of the participants was assessed with the single nucleotide polymorphism (SNPs) test (Lim et al., 2011). The analyses presented in this study were based on a sample of 207 monozygotic twin pairs and 72 same-sex dizygotic twin pairs. A lower number of DZ compared to MZ pairs is commonly found in twin research, and is usually attributed to volunteer bias (Lykken, Tellegen, & Derubeis, 1978). However, there is wide variation in the ratio of live-born MZ to same-sex DZ twins, with some surveys finding especially low rates in Asian populations. The proportion of DZ twins in our research (25.8%) was comparable to the population prevalence reported by Chia et al. (2004) in Singapore (i.e., 24.7% after excluding opposite-sex DZ pairs). The mean age of the MZ twins was 6 years 10 months (*SD* = 1 year 10 months) and that of DZ twins was 6 years 7 months (*SD* = 1 year 9 months). The youngest participant was 3 years 6 months old, while the oldest was 11 years old. The percentages of children in each year band from 3 to 11 years were 3.9%, 17.6%, 18.6%, 14.7%, 15.8%, 15.4%, 7.5%, and 6.5%, respectively. As shown by these figures, most children fell between 4 and 9 years old.

Parental consent was obtained for each of the child participants. The testing was conducted at the children’s school or home by trained experimenters. To ensure that the participants’ listening performance was not confounded by hearing loss, a pure-tone hearing test was administered before the testing session. The tasks were administered in a fixed sequence. In order to minimize the effect of fatigue, participants were instructed to take a rest whenever they wanted to. The whole testing session lasted for 1.5 to 2 hr.

### Measures

All tasks described below had both English and Chinese versions. All measures were self-developed or adapted from standardized tests, and have been used and validated in previous studies (Ho, Leung, & Cheung, 2011; McBride-Chang & Ho, 2005). We conducted a pilot test that measured the performance of 15 children at each grade level from kindergarten Grade 2 to primary school Grade 4 (aged from 4 to 10) in order to get information for arranging test items according to their difficulty level. Also, to ensure the test results were reliable, we included practice items in each task, and children were given feedback by the experimenters in these practice trials.

#### Visual word recognition

In both English and Chinese tests, children were instructed to read aloud a list of words one by one. The items had been selected to ensure an adequate range of difficulty to avoid floor and ceiling effects. The 85 words in the English test were adopted from a written word corpus that incorporated several authorized textbooks used in Hong Kong. These 85 words were arranged in ascending order of difficulty, and the first 49 items were divided into five sets of 8–11 words. Basal and ceiling rules were created according to the pilot test results so as to minimize administration time. The test began at an entry level corresponding to the child’s grade level. The testing proceeded if less than three errors were committed in a set. Otherwise, the tester moved to a set of lower difficulty or terminated testing when the child failed 15 consecutive items. We awarded one mark for each correct response. The Cronbach’s alpha was .99.

The Chinese test consisted of 198 items, with 150 two-character Chinese words adopted from the reading subtest of the Hong Kong Test of Specific Learning Difficulties in Reading and Writing (HKT–SpLD; Ho, Chan, Tsang, & Lee, 2000), a standardized test developed for Hong Kong primary school children. The other 48 items were a list of simple single Chinese characters that were more suitable for kindergarteners. The Cronbach’s alpha of the whole Chinese test was .99.

#### Receptive vocabulary

The two receptive vocabulary tests were selected based on their popularity and similarity in format—both tests used a target picture and three foils. The English vocabulary test was an adaptation of a standardized test, the Receptive One-Word Picture Vocabulary Tests (ROWPVT; Brownell, 2000). The Chinese receptive vocabulary test consisted of 80 items translated and adapted from the Peabody Picture Vocabulary Test (4th ed.; PPVT–IV; Dunn & Dunn, 2007). Different items were used for English and Chinese tests to avoid carryover effects. The correlation between the older version of the ROWPVT (Gardner, 1985) and the PPVT-3 (Dunn & Dunn, 1997) was reported as .79 (Ukrainetz & Blomquist, 2002). On each trial, the child was asked to listen to a word presented orally by the experimenter and then point to the corresponding picture out of four options. The number of items of the English and Chinese test was 39 and 80, respectively, with good internal consistency (Cronbach’s alphas = .92 and .96, respectively). There were separate entry points tied to grade levels and a basal rule that required nine or all trials in the first 10 consecutive trials from the entry point.

#### Phonological awareness

The English and Chinese phonological tests measured children’s abilities in manipulating syllables and identifying subsyllabic phonological units. There were some differences in the two tasks’ formats, which were necessitated by differences between the languages in phonological structure in relation to literacy. The English test consisted of rime detection, syllable deletion, and initial phoneme deletion. The Cronbach’s alpha for the whole English phonological awareness test was .88. In the English rime-detection task, all items were modified items selected from the Alliteration and Rhyme subtest of the Phonological Assessment Battery (Frederickson, Frith, & Reason, 1997). On each trial, we presented three English words to participants and instructed them to identify the two words having the same rime. For example, on the trial, “look/book/horse,” it was “look” and “book” that rhymed. The task started with two sample trials, which were followed by eight test trials. In the English syllable-deletion task, children were required to delete one syllable (at the initial, middle, or final position) from low-frequency three-syllable English words. For example, the experimenter asked the child to repeat orally the word *jamboree* and then mentally remove the middle syllable and say “jamree.” One mark was awarded for each correct response. On each trial of the initial phoneme deletion task, the experimenter read aloud an English word and asked the child to say the word with the initial phoneme taken away (e.g., say “fox” without the initial sound [answer “ox”]).

The Chinese phonological awareness task measured syllable and rhyme awareness. In the Chinese syllable deletion task, children were required to mentally delete one syllable (at the initial, middle, or final position) from a multisyllabic Chinese word orally presented by the experimenter and then say it aloud. There were 15 test items that were a mixture of real words, nonwords, or nonsense words. A real word was a word commonly used in daily life and carried lexical meaning (e.g., 望遠鏡 /*mong6 jyun5 geng3*/[binoculars]). A nonword was a word created by combining random Cantonese syllables that did not carry a lexical meaning as a whole (e.g., 女任綠 /*neoi5 jam6 luk6*/). A nonsense word was a word formed by combining nonsense Cantonese syllables that were phonologically legal but did not exist in written form (e.g., /*fou2 moi1 peng5*/). In the Chinese rime detection task, the child was required to identify the syllable that rhymed with the target syllable among three options. On each trial, the experimenter first orally presented a target syllable (e.g., /*jan4*/) and then read out three syllables (e.g., /*ngaa4*/, /*hau4*/, and /*wan4*/; the correct response is */wan4/*). The Cronbach’s alpha of the whole Chinese phonological awareness test was .88.

#### Phonological memory

The phonological memory span of the children was assessed by nonword repetition tasks. We assessed English phonological memory with the Children’s Test of Nonword Repetition (Gathercole & Baddeley, 1996). Based on the pilot test’s data, we employed 16 test items of English pseudowords with a length of two to five syllables. On each trial, children were required to listen and repeat. The responses were marked by the experimenter on the site and recorded for later rating by a second rater. A similar test of phonological memory was constructed for measuring the skill in Chinese. There were 14 nonword strings constructed by combining two to seven Cantonese syllables that resulted in a meaningless chunk as a whole (e.g., /*dim2 tou2*/). On each trial, the child was required to precisely repeat the nonword string presented aurally through headphones. When scoring a response, a point was awarded separately for each correct syllable and each correct order of two consecutive syllables. One point was deducted for each redundant syllable. For example, a correct response of a five-syllable nonword yielded a score of nine (5 points for correct pronunciation; 4 points for correct order). The inter-rater reliability indicated by the intraclass correlation coefficient was .82. The Cronbach’s alphas of the English and Chinese test were .87 and .90, respectively.

#### Speech discrimination

This task assessed children’s ability to distinguish English and Chinese minimal pairs that differed in one phoneme. We used an AXB speech discrimination task that was presented in the form of a computer game (Bishop, Adams, Nation, & Rosen, 2005). On each trial, three words were presented aurally with the animation of jumping owls. Each owl represented a word and jumped when its corresponding word was presented. Then, children were instructed to indicate whether the leftmost or the rightmost word was the same as a target word located at the middle position. If the child made a correct response, a cartoon picture would appear on the screen; otherwise, a “sigh” sound would be presented. The minimal pairs differed in either the place of articulation or the manner of articulation, or both. Results from the pilot testing showed a ceiling effect in some advanced learners, and therefore we added speechlike noise to the word tokens with a signal-to-noise ratio of –12 dB. Following the six trial items, 24 test items were presented. The Cronbach’s alphas for the English and Chinese versions of the test were .72 and .80, respectively.

## Results

The overall performances of the twin sample are presented in Table 1. Given the wide age range, it was important to adjust scores so that they represented ability relative to age. First, the relations between age and the raw scores on all tasks were examined using regression curve fitting analyses. It was evident that improvement of scores with age was more rapid at younger than older ages, and the best fitting regression of raw scores on age included quadratic and cubic terms, reflecting the nonlinear association, *R*^2^s = .20–.65, *F*s(3, 588) = 45.74–264.34, *p* < .001. The regression equation with age, age^2, and age^3 terms as predictors was used to convert all scores to standardized residuals.[Table-anchor tbl1]

We first computed the intraclass correlations (ICCs) for MZ and DZ twins, respectively (see Table 2). If the ICC of MZ is higher than that of DZ, a genetic effect is implicated. Next, the relative contributions of additive genetic effects (A), shared environmental effects (C), and nonshared environmental effects (E) were computed for all the Chinese and English measures through ACE model fitting (Rijsdijk & Sham, 2002). These analyses were performed using OpenMx structural equation modeling using the R statistical package (Boker et al., 2011). Overall, the models provided satisfactory goodness-of-fit (*p*s > .05). The univariate estimates and the 95% confidence intervals (CI) are presented in Table 3. Power calculation with a significance level α of .05 was conducted for each heritability and shared environmental estimate using Mx (Neale, 1997). For each test, we first submitted the values of A, C and E estimates to generate an expected covariance matrix (the true model). Then, we constrained one pathway (either A or E) and ran a false (restricted) model that yielded a noncentrality χ^2^ (Posthuma & Boomsma, 2005). If the CIs of Chinese and ESL estimates did not overlap, the two estimates were significantly different.[Table-anchor tbl2][Table-anchor tbl3]

For visual word recognition, the genetic influences were strong in both languages (a^2^ = .53 in ESL; .76 in Chinese; power = 1.0). We found moderate shared environmental effects in ESL (c^2^ = .38; power = .14) but negligible shared environmental effects in Chinese. Regarding receptive vocabulary, the genetic influences were low in ESL (a^2^ = .13; power = .50) and negligible in Chinese. Substantial shared environmental influences were found in both ESL and Chinese (c^2^ = .74 in ESL and .56 in Chinese; power = 1.0 and .90, respectively). The pattern of results was very different in the two languages for phonological awareness. Strong heritability was found in ESL (a^2^ = .57; power = .90), but nonsignificant genetic influences were indicated in Chinese. In contrast, shared environmental influences were significant and strong in Chinese (c^2^ = .52; power = .90) but negligible in ESL. For phonological memory, modest to moderate genetic and shared environmental effects were estimated in ESL (a^2^ = .36; c^2^ = .29; power = .38 and .50, respectively). Substantial genetic effects (a^2^ = .72; power = 1.0) and negligible shared environmental influences were indicated in Chinese. Last, the results of speech discrimination indicated a moderate genetic effect in Chinese only (a^2^ = .31, power = .50). This was the only measure that shows substantial nonshared environmental effects in both languages (e^2^ = .72 in ESL and .69 in Chinese). Although the internal reliabilities of the Chinese and ESL tests were not particularly low, the nonshared environmental effects should be interpreted with caution because this component included measurement error.

**Figure 1 fig1:**
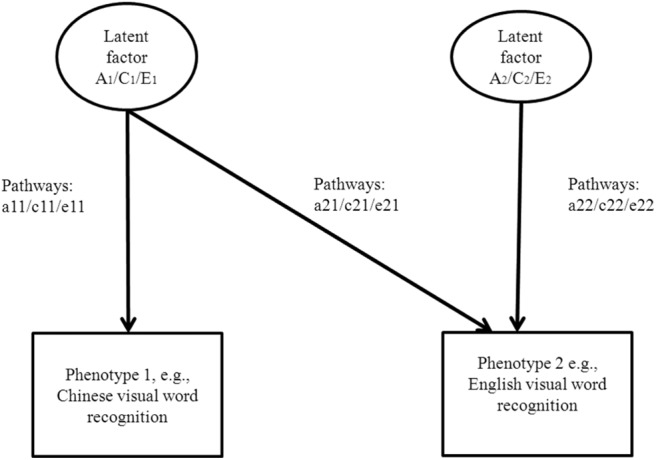
A graphical presentation of the bivariate twin analysis by the Cholesky decomposition method. A/a = additive genetic effects; C/c = shared environmental effects; E/e = nonshared environmental effects.

The results of partial correlation controlling for the effect of age showed that the ESL–Chinese variable pairs were positively and moderately correlated with each other (see Table 4). Initial inspection showed that cross-twin cross-trait correlations of MZ twins tended to be larger than that of DZ twins, suggesting that genetic factors were likely to have an influence on the phenotypic correlations between Chinese and ESL variables. Cholesky decomposition was used to quantify the genetic and environmental overlaps between five Chinese and English variable pairs (e.g., Chinese and ESL visual word recognition). Because the native language develops prior to a second language, in the bivariate analyses, Chinese measures were entered first, followed by ESL measures (see Figure 1). The first set of additive genetic (A), shared environmental (C), and nonshared environmental (E) factors—A1, C1, E1—accounts for the variance in Chinese variables and the covariance between Chinese and ESL variables. The second set—A2, C2, E2—accounts for the remaining variance in ESL variables.[Table-anchor tbl4][Fig-anchor fig1]

Results indicated significant paths linking A1 to each of the Chinese–ESL variable pairs, indicating the common genetic origins of parallel language skills across Chinese and ESL (see Table 5). The genetic correlations among the Chinese–ESL variable pairs were substantial, ranging from .90 to 1.00 (see Table 6). In principle, the degree of genetic overlap between two skills does not rely on the size of univariate estimates of the each of the skills. However, our finding of low heritability in Chinese and ESL receptive vocabulary might suggest an overestimation of the genetic correlation between the Chinese and English parallel skills.[Table-anchor tbl5][Table-anchor tbl6]

Shared environmental overlaps were found in receptive vocabulary and phonological awareness only, as indicated by the significant C1 paths. Shared environmental correlations were .24 and 1.00 for receptive vocabulary and phonological awareness, respectively. Nonshared environmental overlaps were indicated in visual word recognition and speech discrimination only, as indicated by the significant E1 paths, and their nonshared environmental correlations are modest. Power calculation with a significance level α of .05 was conducted by following a principle similar to that used for univariate heritability estimates. The genetic variances and covariances were submitted to generate a true model that was contrasted against a nested model to obtain a noncentrality parameter (Schmitz, Cherny, & Fulker, 1998). Our results showed that the power for rejecting the null hypothesis of genetic/environmental correlation being 0 was above .80, except for the shared environmental correlation between Chinese and ESL receptive vocabulary (.42).

Bivariate heritability, the extent to which the observed phenotypic correlation is influenced by genetic or environmental correlation, was computed for each pair of variables. This index is obtained by multiplying the square root of the Chinese univariate estimate, the square root of the ESL univariate estimate, and the genetic correlation of the ESL–Chinese variable. Results showed that genetic factors explained 15%–53% of the phenotypic correlations between all the ESL–Chinese variable pairs. Shared environmental effects contributed to 15% of the phenotypic correlations between ESL–Chinese receptive vocabulary as well as 24% of those between ESL–Chinese phonological awareness. In addition, nonshared environmental effects, which included measurement errors, explained the phenotypic link in ESL–Chinese speech discrimination (19%).

## Discussion

The present twin study of second language reading acquisition has demonstrated moderate to substantial genetic effects on ESL visual word recognition, phonological awareness, and phonological memory. Comparing the estimates for the two languages, genetic factors play a major role in ESL phonological awareness, while shared environmental factors are more important in contributing to Chinese phonological awareness. Another interesting finding is that strong genetic overlaps were found in parallel language and reading skills across ESL and Chinese. The phenotypic correlations between parallel skills across ESL and Chinese were significantly explained by genetic factors.

This study demonstrated strong genetic effects on ESL and Chinese visual word recognition in Chinese children. These findings were consistent with past twin studies on children speaking alphabetic languages (e.g., Byrne et al., 2002; Dionne, Dale, Boivin, & Plomin, 2003). Also, these findings suggested similar patterns of relative genetic and environmental contributions to word recognition skills in two cross-language levels: across (a) the first or the second language acquisition of English reading as well as (b) across Chinese and English.

Difference in the findings in ESL and Chinese skills were also indicated. First, significant shared environmental effects were found in ESL visual word recognition but not in Chinese visual word recognition, confirming the important role of a common environment in shaping the English skills of our Hong Kong Chinese sample. It is possible that variation in the type and amount of English instruction is a factor to be considered in accounting for this finding. Hong Kong Chinese children are exposed to a non-total-immersion English environment where the use of English is limited in daily life (Lo & Murphy, 2010). For children studying in Chinese as the medium of instruction (CMI) primary schools, they rarely learn and use English outside the English classroom. While schools are provided with guidelines for English instruction, individual schools can develop their own curriculum and activities (Hong Kong Curriculum Development Council, 2004, pp. 7 and 96). Furthermore, more affluent families can provide their children with additional support such as private tutoring by native English speakers or foreign domestic helpers (T. Y. Chan & McBride-Chang, 2005). As reading skills require explicit teaching, these variations in ESL exposure could enhance the impact of environmental factors on ESL word reading skills.

Second, genetic factors play a major role in ESL phonological awareness, while shared environmental factors had a greater contribution in Chinese phonological awareness. This finding is particularly interesting, as it showed a different pattern compared to visual word recognition and phonological memory. As environmental influence is especially sensitive to sample size, which was marginally large enough in our study, the interpretation of data requires caution. However, we may speculate that different phonological training in Chinese and English may affect the relative genetic and environmental contributions. As argued above, where environmental experiences are very different, one is more likely to see significant environmental effects. In Hong Kong, students learn to read Chinese through two spoken languages, namely Cantonese and Mandarin, in Chinese lessons. While the look-and-say method is used in Cantonese classes, Chinese pinyin (a phonetically transparent form of writing) is used during Mandarin classes. There is no standardization of the spoken language used for teaching Chinese words. Therefore, learners in Hong Kong have more diverse orthographic and spoken language experience, which has been shown to produce a long-term effect on the performance in phonological awareness (Cheung, Chen, Lai, Wong, & Hills, 2001). In contrast, given the more regular grapheme–phoneme mappings in English as an alphabetic script, more standardized phonological training is used in English instruction. Thus, the larger environmental variation across schools in Chinese phonetic training might explain the pattern of results.

Third, genetic factors played a more important role in Chinese phonological memory compared to that in ESL. Inspired by the findings of the International Longitudinal Twin Study that showed the influence of reading instruction on the estimate of heritability of reading (Samuelsson et al., 2007), we suspect this result may be related to variations in the extent to which children have comparable environmental exposure to the two languages. In Hong Kong, spoken Chinese is widely used in daily life while spoken English is not. Therefore, exposure to spoken Chinese in daily life is more equal among the children, and any variance in Chinese phonological memory is more likely to reflect genetic factors. In contrast, exposure to English in Hong Kong is dependent on school environment, which varies greatly across the children, as discussed above. For example, teachers in some schools give English dictation to students (i.e., they expect children to write down sentences they utter, as they believe this will strengthen phonological memory). Variations in school practice may contribute to the significant shared environmental influence on ESL phonological memory detected in our study.

The significant genetic correlations and bivariate heritability estimates showed that there were genetic overlaps between parallel Chinese and ESL reading skills. An especially notable finding was that a high degree of genetic overlap and substantial genetic contribution to the phenotypic link between the two languages were evidenced for visual word recognition and phonological memory. In other words, despite the cross-linguistic difference in Chinese and English orthography, the genes that influence the development of Chinese word recognition and phonological memory play a congruent role in the development of the parallel skills in English. These new findings suggested that the *central processing hypothesis* (Gholamain & Geva, 1999) or overlapping brain regions (Perfetti et al., 2007) that were thought to subsume parallel L1 and L2 skills could be explained at the level of genes. Practically speaking, the strong L1–L2 genetic relations imply that children at risk for dyslexia in L1 might also face similar problems in L2 reading (Chung & Ho, 2010). Genetic overlaps and moderate to strong genetic contributions to the cross-linguistic phenotypic links were also found for receptive vocabulary, phonological awareness, and speech discrimination, although these effects were not as strong as in visual word recognition and phonological memory. Our results have provided a basis for further examination of the overlap between parallel Chinese and ESL skills at the molecular genetic level.

Common shared environmental origins across Chinese and ESL were found in receptive vocabulary and phonological awareness. These common environmental influences could be aspects of home literacy environment, such as parent–child reading and parental instructions. For instance, parent–child reading can enhance both Chinese and English phonological awareness in Chinese ESL children (Chow, McBride-Chang, & Cheung, 2010).

### Limitations and Future Directions

There are three major limitations in this study. First, although the sample size we obtained fulfilled the minimal requirement for statistical power, it limited our ability to construct models with more parameters to estimate. In particular, the imbalance of MZ and DZ twins in our present sample limits the power of the study. Rather than recruitment through schools and promotion posters, more systematic sampling procedures to increase the proportion of DZ recruits could be warranted, possibly through the establishment of a twin registry in Hong Kong and Greater China. Second, the age range of participants was relatively large. Although age effects had been controlled for in analyses in this study, we could not rule out the possibility that ACE estimates might vary for different age groups, and a larger longitudinal study would be needed to test that possibility. Third, there was only one measure for each of the constructs. We included the most reliable measure for each of the constructs, but measurement error could not be entirely partialed out. Use of other measures would also render it possible to dissociate overlaps in English–Chinese scores that reflect ability to cope with specific task demands, such as selecting from a multiple choice array. Apart from improving the sampling method and measures, future twin studies of second language learning would extend from the investigation of word reading to sentence reading.

### Conclusion

To conclude, this study shows that learning to read in a second language does not interfere with first language acquisition as we originally expected. On the contrary, but consistent with Geva’s (1999) view, it may help children to abstract general concepts, such as the phonological structure of words, which facilitates learning in both languages. While Coventry et al. (2012) and Dale et al. (2010) have shown genetic overlaps between the first and a second language using questionnaire data, our study has further shown the genetic links between specific reading and related skills in first and second languages by direct measures of children’s skills. With the pair of languages examined being English and Chinese, this study also provides new gene–environment evidence regarding alphabetic and nonalphabetic languages.

## Figures and Tables

**Table 1 tbl1:** Means and Standard Deviations for Raw Scores of English and Chinese Measures Prior to Age Correction (N = 558)

Variable	Mean (*SD*)
English as a second language	
Visual word recognition (max = 85)	34.78 (31.17)
Receptive vocabulary (max = 39)	22.21 (8.06)
Phonological awareness (max = 20)	10.62 (4.95)
Phonological memory (max = 96)	63.83 (19.54)
Speech discrimination (max = 24)	17.34 (3.62)
Chinese	
Visual word recognition (max = 198)	85.39 (62.25)
Receptive vocabulary (max = 80)	53.47 (16.74)
Phonological awareness (max = 24)	15.59 (5.62)
Phonological memory (max = 124)	75.41 (26.75)
Speech discrimination (max = 24)	18.58 (4.19)
*Note.* max = maximum.	

**Table 2 tbl2:** Summary of Intraclass Correlation Coefficients (MZ Twin = 207 Pairs, DZ Twin = 72 Pairs)

	MZ twin	DZ twin
ESL variable		
Visual word recognition	.90 [.88, .93]	.61 [.44, .73]
Receptive vocabulary	.86 [.82, .89]	.80 [.69, .87]
Phonological awareness	.67 [.59, .74]	.40 [.19, .57]
Phonological memory	.62 [.53, .70]	.50 [.31, .66]
Speech discrimination	.26 [.13, .38]	.16 [−.06, .38]
Chinese variable		
Visual word recognition	.89 [.86, .92]	.52 [.33, .67]
Receptive vocabulary	.66 [.57, .73]	.63 [.47, .75]
Phonological awareness	.60 [.51, .68]	.61 [.44, .73]
Phonological memory	.74 [.67, .79]	.43 [.22, .60]
Speech discrimination	.33 [.20, .44]	−.04 [−.27, .18]
*Note.* The 95% confidence intervals for intraclass correlation coefficients are in brackets. MZ = monozygotic; DZ = dizygotic; ESL = English as a second language.		

**Table 3 tbl3:** Univariate ACE Models Fit and Parameter Estimates for All the Hypothesized Variables (MZ = 207 Pairs, DZ = 72 pairs)

Variable	χ^2^	AIC	a^2^	c^2^	e^2^
ESL visual word recognition	8.81	74.11	.53 [.35, .68]	.38 [.19, .57]	.09 [.08, .10]
Chinese visual word recognition	5.26	113.05	.76 [.53, .96]	.14 [−.07, .37]	.10 [.08, .11]
ESL receptive vocabulary	9.26	113.26	.13 [.02, .22]	.74 [.59, .87]	.13 [.11, .15]
Chinese receptive vocabulary	7.10	315.97	.11 [−.08, .30]	.56 [.36, .75]	.33 [.28, .37]
ESL phonological awareness	3.52	332.73	.57 [.28, .83]	.11 [−.14, .38]	.32 [.27, .36]
Chinese phonological awareness	9.64	342.74	.10 [−.11, .30]	.52 [.32, .72]	.38 [.32, .43]
ESL phonological memory	7.67	345.83	.36 [.12, .60]	.29 [.05, .50]	.35 [.30, .40]
Chinese phonological memory	1.65	292.73	.72 [.45, .96]	.04 [−.02, .28]	.24 [.21, .28]
ESL speech discrimination	5.96	455.03	.27 [−.07, .59]	.01 [−.27, .30]	.72 [.64, .83]
Chinese speech discrimination	9.98	452.11	.31 [.20, .41]	0 [0, 0]	.69 [.59, .78]
*Note.* Data in brackets are 95% confidence intervals. The ACE models had a satisfactory goodness-of-fit indicated by a nonsignificant chi-square change between the saturated and the ACE models (chi-square change ranged from 1.65 to 9.98, with a change in degrees of freedom of 6, *p*s > .05). A = additive genetic effects; C = shared environmental effects; E = nonshared environmental effects; MZ = monozygotic; DZ = dizygotic; AIC = Akaike’s information criterion; a^2^ = additive genetic estimates; c^2^ = shared environmental estimates; e^2^ = nonshared environmental estimates; ESL = English as a second language.					

**Table 4 tbl4:** Phenotypic and Cross-Twin Cross-Trait Correlations Between Chinese and ESL Parallel Measures

Chinese and parallel English skills	Phenotypic correlation^a^	MZ Twin 1 ESL skill Twin 2 Chi. skill	DZ Twin 2 ESL skill Twin 1 Chi. skill	MZ Twin 1 Chi. skill Twin 2 ESL skill	DZ Twin 2 Chi. Skill Twin 1 ESL skill
Visual word recognition	.55	.46 [.34, .56]	.43 [.23, .60]	.46 [.35, .56]	.33 [.11, .52]
Receptive vocabulary	.32	.28 [.15, .40]	.19 [−.03, .40]	.35 [.22, .46]	.16 [−.06, .38]
Phonological awareness	.49	.50 [.39, .60]	.37 [.15, .55]	.56 [.46, .64]	.41 [.20, .58]
Phonological memory	.37	.41 [.29, .51]	.10 [−.12, .33]	.52 [.41, .61]	.30 [.08, .50]
Speech discrimination	.52	.22 [.09, .34]	.17 [−.05, .39]	.39 [.27, .50]	.30 [−.11, .56]
*Note.* The 95% confidence interval for correlation coefficients are in brackets. ESL = English as a second language; MZ = monozygotic; DZ = dizygotic; Chi. = Chinese.					
^a^ Data are based on two subsamples created by randomly selected one twin from a twin pair.					

**Table 5 tbl5:** Standardized Unsquared Path Coefficients From Bivariate Cholesky Decomposition of Additive Genetic (A), Shared Environment (C), and Nonshared Environment (E) Correlations Between Chinese and ESL Variables

Variable	A1	A2	C1	C2	E1	E2
CVWR	.85 [.67, 1.02]		.40 [.02, .78]		.31 [.28, .34]	
EVWR	.63 [.46, .80]	.30 [.11, .49]	−.04 [−.66, .56]	.63 [.40, .87]	.07 [.03, .11]	.29 [.26, .32]
CRV	.40 [.12, .67]		.71 [.55, .88]		.57 [.52, .62]	
ERV	.37 [.19, .55]	0 [−.62, .62]	.21 [.02, .40]	.82 [.73, .92]	.01 [−.02, .06]	.36 [.33, .40]
CPA	.37 [.13, .62]		.69 [.54.85]		.61 [.56, .66]	
EPA	.74 [.59, .88]	0 [−1.31, 1.31]	.36 [.10, .62]	0 [−1.50, .1.50]	0 [−.07, .07]	.57 [.51, .62]
CPM	.72 [.62, 1.05]		.21 [−.59, 1.01]		.49 [.45, .54]	
EPM	.49 [.31, .85]	.11 [−1.37, 1.6]	−.14 [−1.70, 1.41]	.51 [−.13, 1.16]	−.03 [−.11, .05]	.59 [.54, .65]
CSP	.57 [.45, .69]		0 [−.47, .47]		.81 [.74, .89]	
ESP	.55 [.43, .67]	0 [−.41, .41]	0 [−.66, .66]	0 [−.38, .38]	.24 [.14, .34]	.79 [.73, .85]
*Note.* Data in brackets are 95% confidence intervals. The 1 and 2 in, for example, A1 indicate Latent Variable 1 or Latent Variable 2 (see Figure 1). ESL = English as a second language; CVWR = Chinese visual word recognition; EVWR = ESL visual word recognition; CRV = Chinese receptive vocabulary; ERV = ESL receptive vocabulary; CPA = Chinese phonological awareness; EPA = ESL phonological awareness; CPM = Chinese phonological memory; EPM = ESL phonological memory; CSP = Chinese speech discrimination; ESP = ESL speech discrimination.						

**Table 6 tbl6:** Summary of Two Indices Yielded From Bivariate Twin Analyses of Chinese and ESL Variables

Chinese and parallel English skills	Genetic/environmental correlation			Bivariate heritability		
	A	C	E	A	C	E
Visual word recognition	.90	*ns*	.24	.53	*ns*	.02
Receptive vocabulary	1.00	.24	*ns*	.15	.15	*ns*
Phonological awareness	1.00	1.00	*ns*	.27	.24	*ns*
Phonological memory	.98	*ns*	*ns*	.35	*ns*	*ns*
Speech discrimination	1.00	*ns*	.29	.31	*ns*	.19
*Note.* ESL = English as a second language; A = additive genetic factor; C = shared environmental factor; E = nonshared environmental factor; *ns* = nonsignificant pathway.						

## References

[c1] Bialystok E., McBride-Chang C., & Luk G. (2005). Bilingualism, language proficiency, and learning to read in two writing systems. Journal of Educational Psychology, 97, 580–590. doi:10.1037/0022-0663.97.4.580

[c2] Bishop D. V. M., Adams C. V., Nation K., & Rosen S. (2005). Perception of transient nonspeech stimuli is normal in specific language impairment: Evidence from glide discrimination. Applied Psycholinguistics, 26, 175–194. doi:10.1017/S0142716405050137

[c3] Boets B., Wouters J., Van Wieringen A., De Smedt B., & Ghesquiere P. (2008). Modelling relations between sensory processing, speech perception, orthographic and phonological ability, and literacy achievement. Brain and Language, 106, 29–40. doi:10.1016/j.bandl.2007.12.004 18207564

[c4] Boker S., Neale M., Maes H., Wilde M., Spiegel M., Brick T., . . . Fox J. (2011). OpenMx: An open source extended structural equation modeling framework. Psychometrika, 76, 306–317. doi:10.1007/s11336-010-9200-6 23258944 PMC3525063

[c5] Brown C. (2000). The interrelation between speech perception and phonological acquisition from infant to adult In Archibald J. (Ed.), Second language acquisition and linguistic theory (pp. 4–63). Malden, MA: Blackwell.

[c6] Brownell R. (2000). Receptive one-word picture vocabulary test (2nd ed.). Novato, CA: Academic Therapy.

[c7] Bryant P. E., Maclean M., & Bradley L. L. (1990). Rhyme, language, and children’s reading. Applied Psycholinguistics, 11, 237–252. doi:10.1017/S0142716400008870

[c8] Byrne B., Delaland C., Fielding-Barnsley R., Quain P., Samuelsson S., Høien T., . . . Olson R. K. (2002). Longitudinal twin study of early reading development in three countries: Preliminary results. Annals of Dyslexia, 52, 47–73. doi:10.1007/s11881-002-0006-9

[c9] Chan A. Y. W. (2006). Cantonese ESL learners’ pronunciation of English final consonants. Language, Culture and Curriculum, 19, 296–313. doi:10.1080/07908310608668769

[c10] Chan T. Y., & McBride-Chang C. (2005). Environment and bilingualism in Hong Kong kindergartners: The impact of foreign domestic helpers on early language-learning. Journal of Psychology in Chinese Societies, 6, 179–193.

[c11] Cheung H., Chen H.-C., Lai C. Y., Wong O. C., & Hills M. (2001). The development of phonological awareness: Effects of spoken language experience and orthography. Cognition, 81, 227–241. doi:10.1016/S0010-0277(01)00136-6 11483171

[c12] Cheung H., Chung K. K. H., Wong S. W. L., McBride-Chang C., Penney T. B., & Ho C. S.-H. (2010). Speech perception, metalinguistic awareness, reading, and vocabulary in Chinese-English bilingual children. Journal of Educational Psychology, 102, 367–380. doi:10.1037/a0017850

[c13] Chia K. S., Lee J. J. M., Cheung P., Cheung K. H., Seielstad M., Wilcox M. M., & Liu E. (2004). Twin birth in Singapore: A population-based study using the national birth registry. Annals Academy of Medicine Singapore, 33, 195–199. 15098633

[c14] Chiappe P., Glaeser B., & Ferko D. (2007). Speech perception, vocabulary, and the development of reading skills in English among Korean- and English-speaking children. Journal of Educational Psychology, 99, 154–166. doi:10.1037/0022-0663.99.1.154

[c15] Chow B. W. Y., Ho C. S.-H., Wong S. W. L., Waye M. M. Y., & Bishop D. V. M. (2013). Generalist genes and cognitive abilities in Chinese twins. Developmental Science, 16, 260–268. doi:10.1111/desc.12022 23432835 PMC3757317

[c16] Chow B. W.-Y., McBride-Chang C., & Cheung H. (2010). Parent-child reading in English as a second language: Effects on language and literacy development of Chinese kindergarteners. Journal of Research in Reading, 33, 284–301. doi:10.1111/j.1467-9817.2009.01414.x

[c17] Chung K. K. H., & Ho C. S. H. (2010). Second language learning difficulties in Chinese children with dyslexia: What are the reading-related cognitive skills that contribute to English and Chinese word reading? Journal of Learning Disabilities, 43, 195–211. doi:10.1177/0022219409345018 19897734

[c18] Coventry W., Antón-Méndez I., Ellis E. M., Levisen C., Byrne B., van Daal V. H., & Ellis N. C. (2012). The etiology of individual differences in second language acquisition in Australian school students: A behavior genetic study. Language Learning, 62, 880–901. doi:10.1111/j.1467-9922.2012.00718.x

[c19] Dale P. S., Harlaar N., Haworth C. M. A., & Plomin R. (2010). Two by two: A twin study of second-language acquisition. Psychological Science, 21, 635–640. doi:10.1177/0956797610368060 20483839

[c20] Dionne G., Dale P. S., Boivin M., & Plomin R. (2003). Genetic evidence for bidirectional effects of early lexical and grammatical development. Child Development, 74, 394–412. doi:10.1111/1467-8624.7402005 12705562

[c102] Dunn L. M., & Dunn L. M. (1997). PPVT-3: Peabody Picture Vocabulary Test: Third edition. Circle Pines, MN: American Guidance Service.

[c21] Dunn L. M., & Dunn D. M. (2007). PPVT-4: Peabody Picture Vocabulary Test: Fourth edition. Minneapolis, MN: NCS Pearson.

[c22] Frederickson N., Frith U., & Reason R. (1997). Phonological assessment battery: Standardisation edition. Windsor, England: NFER-Nelson.

[c101] Gardner M. F. (1985). Receptive One-Word Picture Vocabulary Test. Novato, CA: Academic Therapy.

[c23] Gathercole S. E., & Baddeley A. D. (1996). Children’s test of nonword repetition. London, England: Pearson Assessment. 10.1080/09658219408258940 7584287

[c24] Gathercole S. E., Hitch G. J., Service E., & Martin A. J. (1997). Phonological short-term memory and new word learning in children. Developmental Psychology, 33, 966–979. doi:10.1037/0012-1649.33.6.966 9383619

[c25] Genesee F., Geva E., Dressler C., & Kamil M. (2006). Synthesis: Cross-linguistic relationships In August D. & Shanahan T. (Eds.), Report of the national literacy panel on language minority youth and children (pp. 153–174). Mahwah, NJ: Erlbaum.

[c26] Geva E. (1999). Issues in the development of second language reading: Implications for instruction and assessment In Nunes T. (Ed.), Learning to read: An integrated view from research and practice (pp. 343–367). Dordrecht, the Netherlands: Kluwer Academia. doi:10.1007/978-94-011-4826-9_20

[c27] Gholamain M., & Geva E. (1999). Orthographic and cognitive factors in the concurrent development of basic reading skills in English and Persian. Language Learning, 49, 183–217. doi:10.1111/0023-8333.00087

[c28] Graddol D. (2006). English next: Why global English may mean the end of “English as a foreign language.” London, England: British Council.

[c29] Ho C. S.-H., Chan D. W., Tsang S., & Lee S. (2000). 香港讀寫障礙測驗 [The Hong Kong Test of Specific Learning Difficulties in Reading and Writing (HKT-SpLD) manual]. Hong Kong, China: Hong Kong Specific Learning Difficulties Research Team.

[c30] Ho C. S.-H., Leung M.-T., & Cheung H. (2011). Early difficulties of Chinese preschoolers at familial risk for dyslexia: Deficits in oral language, phonological processing skills, and print-related skills. Dyslexia, 17, 143–164. doi:10.1002/dys.429 21294232

[c45] Hong Kong Curriculum Development Council (2004). English language curriculum guide (Primary 1–6). Retrieved from http://www.edb.gov.hk/attachment/en/curriculum-development/doc-reports/guide-kla-gs-primary-curriculum/cdc_ele_kla_curriculum_guide_(p1-s3)_2002.pdf

[c31] Lim C. K. B., Yeung V. S. Y., Yeung T. L., Tam A. C. Y., Ho C. S.-H., Wong S. W. L., . . . Waye M. M. Y. (2011). Genotype analyses using SNP (using MALDI-TOF mass spectrometry) and STR (microsatellite) markers in the determination of zygosity status of Chinese twins. Journal of Biochemistry and Molecular Biology in the Post Genomic Era, 1, 51–64.

[c32] Lo Y. Y., & Murphy V. A. (2010). Vocabulary knowledge and growth in Immersion and Regular Language Learning Programmes in Hong Kong. Language and Education, 24, 215–238. doi:10.1080/09500780903576125

[c33] Lykken D. T., Tellegen A., & Derubeis R. (1978). Volunteer bias in twin research: The rule of two-thirds. Biodemography and Social Biology, 25, 1–9. doi:10.1080/19485565.1978.9988312 565949

[c34] McBride-Chang C., & Ho C. S.-H. (2005). Predictors of beginning reading in Chinese and English: A 2-year longitudinal study of Chinese kindergartners. Scientific Studies of Reading, 9, 117–144. doi:10.1207/s1532799xssr0902_2

[c35] McBride-Chang C., Tong X., Shu H., Wong A. M. Y., Leung K., & Tardif T. (2008). Syllable, phoneme, and tone: Psycholinguistic units in early Chinese and English word recognition. Scientific Studies of Reading, 12, 171–194. doi:10.1080/10888430801917290

[c36] Melby-Lervåg M., Lyster S., & Hulme C. (2012). Phonological skills and their role in learning to read: A meta-analytic review. Psychological Bulletin, 138, 322–352. doi:10.1037/a0026744 22250824

[c37] Neale M. C. (1997). Mx: Statistical modelling (4th ed.) Unpublished computer software, Medical College of Virginia, Department of Psychiatry.

[c39] Oakhill J. V., & Cain K. (2012). The precursors of reading ability in young readers: Evidence from a four-year longitudinal study. Scientific Studies of Reading, 16, 91–121. doi:10.1080/10888438.2010.529219

[c40] Perfetti C. A., Liu Y., Fiez J., Nelson J., Bolger D. J., & Tan L.-H. (2007). Reading in two writing systems: Accommodation and assimilation of the brain’s reading network. Bilingualism: Language and Cognition, 10, 131–146. doi:10.1017/S1366728907002891

[c41] Posthuma D., & Boomsma D. I. (2005). Mx Scripts Library: Structural equation modeling scripts for twin and family data. Behavior Genetics, 35, 499–505. doi:10.1007/s10519-005-2791-5 15971030

[c42] Rijsdijk F. V., & Sham P. C. (2002). Analytic approaches to twin data using structural equation models. Briefings in Bioinformatics, 3, 119–133. 12139432 10.1093/bib/3.2.119

[c43] Samuelsson S., Olson R., Wadsworth S., Corley R., DeFries J. C., Willcutt E., . . . Byrne B. (2007). Genetic and environmental influences on prereading skills and early reading and spelling development in the United States, Australia, and Scandinavia. Reading and Writing, 20, 51–75. doi:10.1007/s11145-006-9018-x

[c44] Schmitz S., Cherny S. S., & Fulker D. W. (1998). Increase in power through multivariate analyses. Behavior Genetics, 28, 357–363. doi:10.1023/A:1021669602220 9926617

[c46] Ukrainetz T. A., & Blomquist C. (2002). The criterion validity of four vocabulary tests compared with a language sample. Child Language Teaching & Therapy, 18, 59–78. doi:10.1191/0265659002ct227oa

[c47] Wagner R. K., Torgesen J. K., & Rashotte C. A. (1994). Development of reading-related phonological processing abilities: New evidence of bidirectional causality from a latent variable longitudinal study. Developmental Psychology, 30, 73–87. doi:10.1037/0012-1649.30.1.73

